# Impact of vaccination on hospitalization and mortality from COVID-19 in patients with primary and secondary immunodeficiency: The United Kingdom experience

**DOI:** 10.3389/fimmu.2022.984376

**Published:** 2022-09-23

**Authors:** Adrian M. Shields, Susan Tadros, Adam Al-Hakim, Jeremy M. Nell, Me Me Nay Lin, Michele Chan, Sarah Goddard, John Dempster, Magdalena Dziadzio, Smita Y. Patel, Shuayb Elkalifa, Aarnoud Huissoon, Christopher J. A. Duncan, Archana Herwadkar, Sujoy Khan, Claire Bethune, Suzanne Elcombe, James Thaventhiran, Paul Klenerman, David M. Lowe, Sinisa Savic, Siobhan O. Burns, Alex G. Richter

**Affiliations:** ^1^ Clinical Immunology Service, Institute of Immunology and Immunotherapy, University of Birmingham, Birmingham, United Kingdom; ^2^ Department of Clinical Immunology, University Hospitals Birmingham National Health Service (NHS) Foundation Trust, Birmingham, United Kingdom; ^3^ Department of Immunology, Royal Free London National Health Service (NHS) Foundation Trust, London, United Kingdom; ^4^ Department of Allergy and Clinical Immunology, Leeds Teaching Hospitals National Health Service (NHS) Trust, Leeds, United Kingdom; ^5^ Department of Infection and Tropical Medicine, Newcastle upon Tyne Hospitals National Health Service (NHS) Foundation Trust and Translational and Clinical Research Institute, Newcastle University, Newcastle upon Tyne, United Kingdom; ^6^ Department of Clinical Immunology, University Hospitals North Midlands, Stoke-on-Trent, United Kingdom; ^7^ Department of Clinical Immunology, University College London Hospital National Health Service (NHS) Foundation Trust, London, United Kingdom; ^8^ National Institute for Health and Care Research (NIHR) Biomedical Research Centre (BRC) Oxford Biomedical Centre, University of Oxford, Oxford, United Kingdom; ^9^ Department of Clinical Immunology, Oxford University Hospitals National Health Service (NHS) Foundation Trust, Oxford, United Kingdom; ^10^ Department of Immunology, Salford Royal National Health Service (NHS) Foundation Trust, Salford, United Kingdom; ^11^ Department of Clinical Immunology, Hull University Teaching Hospitals National Health Service (NHS) Trust, Hull, United Kingdom; ^12^ Department of Allergy and Clinical Immunology, University Hospitals Plymouth National Health Service (NHS) Trust, Plymouth, United Kingdom; ^13^ Department of Allergy and Clinical Immunology, Newcastle upon Tyne Hospitals National Health Service (NHS) Foundation Trust, Newcastle upon Tyne, Newcastle, United Kingdom; ^14^ Medical Research Council Toxicology Unit, University of Cambridge, Cambridge, United Kingdom; ^15^ Nuffield Department of Medicine, University of Oxford, Oxford, United Kingdom; ^16^ Institute of Immunity and Transplantation, University College London, London, United Kingdom

**Keywords:** COVID-19, CVID, inborn errors of immunity, primary immunodeficiency, secondary immunodeficiency, vaccination, SARS-CoV-2

## Abstract

**Background:**

Individuals with primary and secondary immunodeficiency (PID/SID) were shown to be at risk of poor outcomes during the early stages of the SARS-CoV-2 pandemic. SARS-CoV-2 vaccines demonstrate reduced immunogenicity in these patients.

**Objectives:**

To understand whether the risk of severe COVID-19 in individuals with PID or SID has changed following the deployment of vaccination and therapeutics in the context of the emergence of novel viral variants of concern.

**Methods:**

The outcomes of two cohorts of patients with PID and SID were compared: the first, infected between March and July 2020, prior to vaccination and treatments, the second after these intervention became available between January 2021 and April 2022.

**Results:**

22.7% of immunodeficient patients have been infected at least once with SARS-CoV-2 since the start of the pandemic, compared to over 70% of the general population. Immunodeficient patients were typically infected later in the pandemic when the B.1.1.529 (Omicron) variant was dominant. This delay was associated with receipt of more vaccine doses and higher pre-infection seroprevalence. Compared to March-July 2020, hospitalization rates (53.3% vs 17.9%, p<0.0001) and mortality (Infection fatality rate 20.0% vs 3.4%, p=0.0003) have significantly reduced for patients with PID but remain elevated compared to the general population. The presence of a serological response to vaccination was associated with a reduced duration of viral detection by PCR in the nasopharynx. Early outpatient treatment with antivirals or monoclonal antibodies reduced hospitalization during the Omicron wave.

**Conclusions:**

Most individuals with immunodeficiency in the United Kingdom remain SARS-CoV-2 infection naïve. Vaccination, widespread availability of outpatient treatments and, possibly, the emergence of the B.1.1.529 variant have led to significant improvements in morbidity and mortality followings SARS-CoV-2 infection since the start of the pandemic. However, individuals with PID and SID remain at significantly increased risk of poor outcomes compared to the general population; mitigation, vaccination and treatment strategies must be optimized to minimize the ongoing burden of the pandemic in these vulnerable cohorts.

## Introduction

The COVID-19 pandemic has disproportionately affected individuals with immunodeficiency (1–3). Although vaccination has uncoupled SARS-CoV-2 infection from severe COVID-19 morbidity and mortality in the general population (4), impaired vaccine responses are observed in patients with primary (PID) and secondary immunodeficiency (SID) (5–10) and the relationship between vaccine immunogenicity and real-world efficacy against severe disease in these groups remains unclear.

We have previously described significantly increased risks of morbidity and mortality following SARS-CoV-2 infection in patients with PID and SID (1). We undertook this study to understand whether the risk and determinants of severe COVID-19 in individuals with PID or SID has evolved following the deployment of vaccinations to prevent, and antiviral medication and monoclonal antibodies to treat severe COVID-19, in the context of emerging SARS-CoV-2 variants of concern.

## Methods

Patients were recruited to this study from the COVID-19 in Antibody Deficiency (COV-AD) study (9, 10), a prospective UK study examining the immunological response to infection and vaccination in patients with primary or secondary antibody deficiency, or the ongoing United Kingdom Primary Immunodeficiency Network (UK PIN) national COVID-19 case series (1).

Recruitment to the COV-AD study has been described in detail elsewhere (9, 10). From March 2021, patients with primary or secondary antibody deficiency were eligible for recruitment if they were i) over 18 years of age and ii) receiving immunoglobulin replacement therapy or had a serum IgG concentration less than 4g/L *and* were receiving regular antibiotic prophylaxis to prevent infections. Patients were followed prospectively after enrolment and serological responses to SARS-CoV-2 vaccination or infection were assessed as previously described (9, 10). In the event of polymerase chain reaction (PCR) proven SARS-CoV-2 infection, participants were invited to submit further nasopharyngeal swabs for SARS-CoV-2 PCR every fortnight until viral clearance, defined by 2 consecutive negative nasopharyngeal swabs. PCR was performed as previously described (11). The COV-AD study was approved by the London - Dulwich Research Ethics Committee (REC reference: 21/LO/0162) and funded by United Kingdom Research and Innovation (MR/W002663/1).

The UK PIN COVID-19 case series began collecting data on the outcomes of SARS-CoV-2 infection in patients under the care of Clinical Immunology teams across the UK in March 2020 – detailed description of the methods used in this data collection are provided elsewhere (1). The UK PIN COVID-19 case series collects fully anonymized, routinely collected data regarding patient outcome; no specific ethical approval is required for this collation or publication in accordance with National Health Service Health Research Authority guidance.

Cohort A has been described previously (1) and was derived exclusively from the UK PIN COVID-19 case series. Cohort A represents patients infected with SARS-CoV-2 in the UK between March and July 2020, prior to the widespread availability of treatments for COVID-19 and prior to the rollout of the UK vaccination program, which began in January 2021, during which patients with immunodeficiency were prioritized to receive early vaccination. Cohort B was derived by combining data from participants enrolled in the COV-AD study, infected with SARS-CoV-2 between January 2021 and April 2022 (n=80 had complete data on symptoms and treatments available, representing 69% of all infections or reinfections occurring in COVAD during this period) with additional individuals (n=75) from the UK PIN COVID-19 case series also infected during this period. These additional participants enabled a greater number of individuals with infection to be studied and allowed fair comparison with Cohort A which documented a broader cohort of immunodeficiency patients than just the antibody deficient patients enrolled in COV-AD.

UK National statistics on the number of SARS-CoV-2 cases, variant prevalence, the number of hospital admissions and the number of deaths from COVID-19 were derived from national, publicly available data collated by the United Kingdom Health Security Agency or the Office of National Statistics (12, 13).

In addition to demographics, hospitalization rates and outcome following infection, the prevalence of the following comorbidities were compared between cohorts A and B: chronic lung disease, cardiovascular disease, rheumatological disease, liver disease, diabetes mellitus, chronic renal disease, organ specific autoimmune disease and chronic gastrointestinal disease. Data on ethnicity and body mass index were not available for all UK PIN COVID-19 case series participants and these variables were not included in further analysis. Data were analyzed using GraphPad Prism 9.0 (GraphPad Prism Software, San Diego, Calif). Differences between categorical variables were evaluated using the 2-tailed Chi-square test, differences between ordinal data were evaluated the Mann Witney U test.

## Results

**Figure 1:**
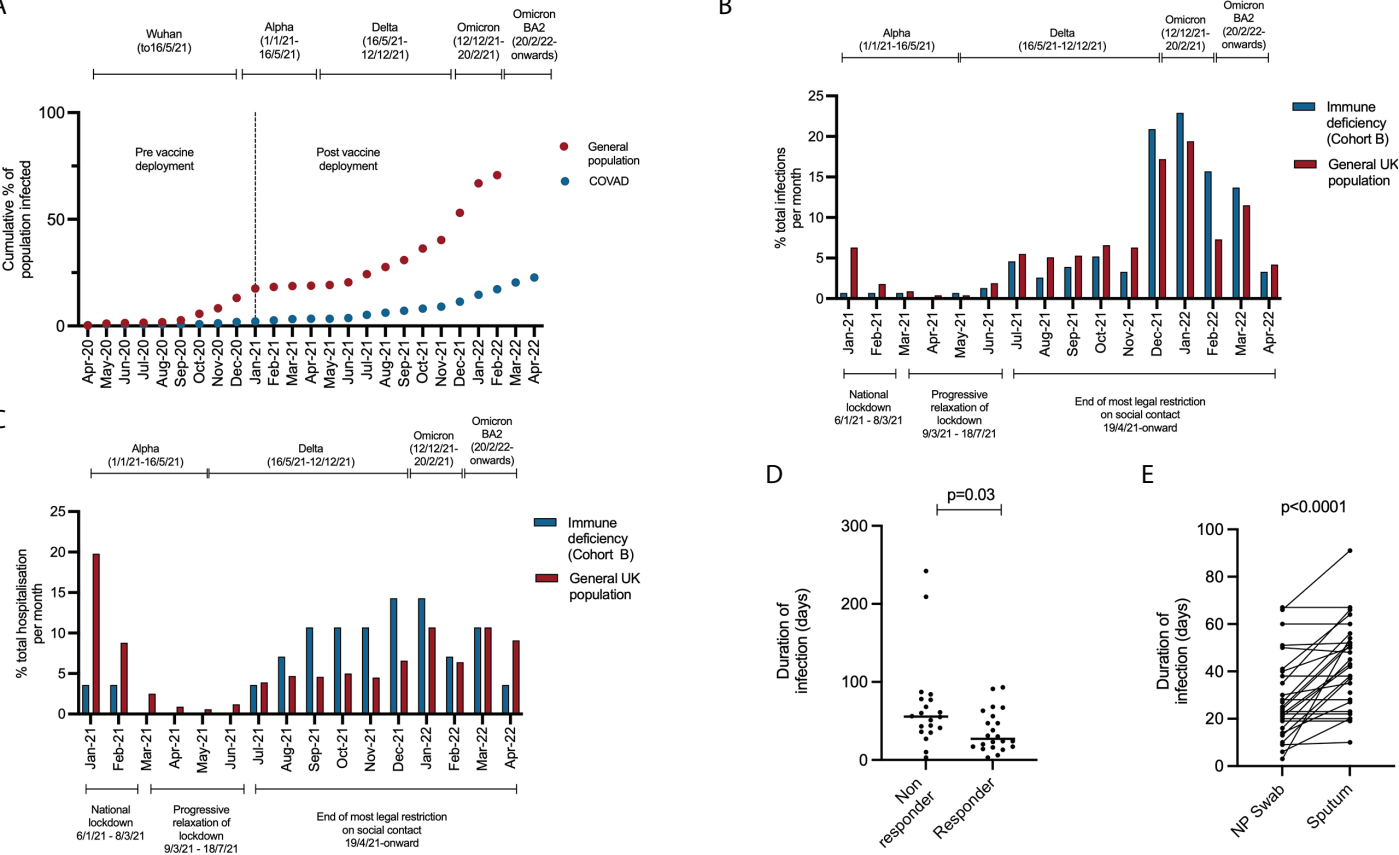
**(A)** Comparison of the cumulative incidence of individuals in the COV-AD study (n = 534) and the general UK population (n = 54,400,000) testing positive for SARS-CoV-2 at least once between April 2020 and April 2022. Data for the UK population is sourced from the UK Office of National Statistics (14). **(B)** Comparison of the timing of SARS-CoV-2 infection in Cohort B compared to the general population between January 2021 and April 2022. Between January 2021 and April 2022 there were 19,379,700 PCR+ SARS-CoV-2 infections in the general population (red bars) and 155 PCR+ SARS-CoV-2 infections in cohort B (blue bars). The proportion of the total number of infections occurring between January 2021 and April 2022 are shown monthly for each group (e.g. January 2021, general population: 1,223,983/19,379,700 infections occurred; 6.3% of all infections between January 2021 and April 2022). **(C)** Comparison of the timing of hospitalization from COVID-19 in Cohort B compared to the general population between January 2021 and April 2022. Between January 2021 and April 2022 there were 571,254 hospitalizations for COVID-19 in the general population (red bars) and 28 hospitalization for COVID-19 in cohort B (blue bars). The proportion of the total number of hospitalization occurring between January 2021 and April 2022 are shown monthly for each group (e.g. January 2021, general population: 113,145/571,254 hospitalization occurred; 19.8% of all hospitalizations between January 2021 and April 2022). **(D)** Comparison of the time to SARS-CoV-2 viral clearance in serological responders (n = 22) and non-responders (n = 21) to prior infection or vaccination. **(E)** Comparison of time to SARS-CoV-2 viral clearance in paired nasopharyngeal and sputum samples.

To define the epidemiology of SARS-CoV-2 infection in patients with immunodeficiency, we report the incidence and timing of SARS-CoV-2 infection in all participants in the COV-AD study (n=534). COV-AD participants had any prior PCR+ infection documented at study enrolment, were screened by PCR for current infection at study entry and remain under longitudinal follow up. As of April 2022, 22.7% (n=121/534) participants had suffered at least one confirmed SARS-CoV-2 infections. By comparison, the UK Office of National Statistics estimated that the cumulative incidence of SARS-CoV-2 infection in the UK general population was 70.7% by February 2022 (**Figure 1A**). Marked divergence in the cumulative incidence of SARS-CoV-2 infection between the general population and COV-AD participants was observed between November and January 2020 (B.1.1.7 - alpha wave), and between July 2021 and February 2022 (late Delta (B.1.617.2) and early Omicron (B.1.1.529) waves).

We have previously reported poor outcomes following SARS-CoV-2 infection in patients with PID and SID in the UK between March and July 2020 (1). To consider the impact of vaccination and therapies on outcome for patients with PID and SID, we compared our original cohort [Cohort A – described in (1)] representing individuals infected prior to SARS-CoV-2 vaccination and the routine use of antiviral and monoclonal antibody therapeutics, outside of the RECOVERY trial (15–17), with a novel cohort of 155 patients infected between January 2021 and April 2022 (Cohort B) after the deployment of these interventions (see Methods for description of recruitment). Cohort A (n=93) included 60 individuals with PID and 33 with SID and Cohort B (n=155) included 117 individuals with PID and 38 with SID (**Table 1**). No significant difference in the prevalence of common co-morbidities that might affect outcome (18), were observed between Cohorts A and B. In cohort B, the mean number of vaccines received at the point of infection was 2.74 for individuals with PID and 2.72 for individuals with SID.

**Table 1:** Demographics and disease characteristics of Cohort B.

	n	Age (yr, IQR)	Sex (% male)	2 vaccine doses (%)	3 vaccine doses (%)	IgRT (%)	Prophylactic antibiotics (pAbx) (%)	Immune suppression (%)	Prior COVID-19 (%)	Symptomatic (%)	Hospitalised (%)	Deaths from COVID-19	Inpatient mortality (%)	IFR (%)
**Primary Immunodeficiency (all)**	**117**	**45 (33-59)**	**52.9**	**92.3**	**81.2**	**83.8**	**51.3**	**16.2**	**16.2**	**85.4**	**17.9**	**4**	**19.0**	**3.42**
22q11microdeletion syndrome	3	22 (22-23)	100.0	100.0	66.7	0.0	100.0	0.0	33.3	100.0	33.3	1	**100.0**	33.33
Autoimmune polyendocrine syndrome-1	1	20s	100.0	0.0	0.0	0.0	0.0	0.0	0.0	100.0	0.0	0	0.0	0.00
CTLA-4 haploinsufficiency	1	50s	100.0	100.0	100.0	100.0	0.0	0.0	0.0	100.0	0.0	0	0.0	0.00
Idiopathic CD4 lymphopenia	2	47.5 (39-56)	0.0	50.0	50.0	0.0	50.0	0.0	0.0	100.0	0.0	0	0.0	0.00
Common variable immunodeficiency	50	45 (37-60.3)	48.0	96.0	86.0	96.0	58.0	22.0	12.0	78.0	20.0	1	**10.0**	2.0
Goods Syndrome	4	69 (52.3-73)	50.0	100.0	100.0	75.0	75.0	25.0	25.0	100.0	0.0	0	0.0	0.00
Hyper IgE syndrome	2	30.5 (25-36)	0.0	100.0	100.0	50.0	100.0	0.0	50.0	100.0	0.0	0	0.0	0.00
Hyper IgM syndrome*	4	32 (22.8-47.3)	75.0	100.0	75.0	100.0	75.0	0.0	25.0	100.0	0.0	0	0.0	0.00
ICOS deficiency	2	46 (39-53)	50.0	50.0	50.0	50.0	0.0	0.0	0.0	100.0	50.0	1	**100.0**	50.00
IgA deficiency	4	32.5 (25.3-55.5)	50.0	75.0	75.0	25.0	25.0	0.0	0.0	75.0	0.0	0	0.0	0.00
IgG subclass deficiency	6	54.0 (33.3-73.0)	50.0	100.0	100.0	83.3	50.0	16.7	16.7	100.0	0.0	0	0.0	0.00
IPEX syndrome	1	Teens	100.0	100.0	100.0	0.0	0.0	100.0	100.0	100.0	0.0	0	0.0	0.00
IRAK-4 deficiency	1	60s	100.0	100.0	100.0	0.0	100.0	0.0	0.0	100.0	0.0	0	0.0	0.00
NFKB1 haploinsufficiency	1	30s	0.0	100.0	100.0	100.0	0.0	0.0	0.0	n/a	0.0	0	0.0	0.00
Primary antibody deficiency	11	52.0 (43-63)	27.2	90.9	90.9	90.9	36.4	27.2	9.1	90.9	27.2	1	33.3	9.09
PNP SCID (post BMT)	2	Teens	50.0	100.0	50.0	100.0	100.0	50.0	0.0	100.0	0.0	0	0.0	0.00
Specific polysaccharide antibody deficiency	6	58.0 (51.3-72.3)	0.0	100.0	100.0	100.0	16.7	16.7	0.0	83.3	0.0	0	0.0	0.00
STAT3 Gain of Function	1	40s	0.0	100.0	100.0	0.0	100.0	0.0	0.0	100.0	0.0	0	0.0	0.00
Undefined combined immunodeficiency	2	47.5 (41-54)	50.0	100.0	100.0	100.0	50.0	0.0	50.0	100.0	50.0	0	0.0	0.00
X-linked agammaglobulinaemia	13	33 (24.5-43)	100.0	92.3	53.8	100.0	30.8	0.0	38.5	84.6	38.5	0	0.0	0.00
**Secondary Immunodeficiency (all)**	**38**	**56.5 (48-64.5)**	**44.7**	**97.4**	**91.8**	**71.1**	**50.0**	**36.8**	**13.2**	**81.6**	**18.4**	**3**	**42.9**	**7.89**

For continuous variables the median and interquartile range is provided. In bold to draw attention to them, they are primary headings for the tables.

The timing of SARS-CoV-2 infections and hospitalizations in cohort B occurring after vaccine deployment from January 2021 differed from the general population (**Figures 1B, C**). In the general population, 9.0% (n=1,736,225/19,379,700) of all infections and 31.2% (n=177,963/571,254) of all hospital admission that occurred between January 2021 and April 2022 happened prior to March 2021 when the B.1.1.7 (alpha) SARS-CoV-2 variant was dominant and prior to widespread vaccination of the general public. In contrast, infections and hospitalization markedly increased in Cohort B after July 2021, coinciding with the relaxation of legal restrictions of social contact. Indeed, 76.5% of all infections in Cohort B (n=155) occurred after November 2021, when the more transmissible B.1.1.529 (omicron) SARS-CoV-2 variant became dominant (19).

Compared to the initial stages of the pandemic, we observed significantly improved outcome for individuals with immunodeficiency (**Table 2**): comparison of Cohort A and Cohort B found reductions in hospitalization rates (53.3% vs 17.9%, p<0.0001) and mortality (Infection fatality rate 20.0% vs 3.4%, p=0.0003) amongst patients with PID. Significant improvements were also seen in patients with CVID (hospitalization rate: 56.5% vs 20.0%, p=0.002, IFR: 34.8% vs 2.0%, p<0.0001) and SID (hospitalization rate: 75.8% vs 18.4%, p<0.0001, IFR: 33.3% vs 7.89%, p<0.007) (**Table 2**). However, hospitalization and mortality in all Cohort B subgroups remained elevated compared to the UK general population [UK general population January 2021 to March 2022: hospitalization rate 2.95%, IFR 0.45% (12)]. Individuals who died in Cohort B tended to have complex disease and co-morbidities that may have contributed to the severity of COVID-19 and, on average, died at a younger age than the general population [average age 58 years vs 71-83 years (20)]. A narrative review of deaths from COVID-19 in Cohort B is provided in **Table 3**.

**Table 2:** Comparison of hospitalization and mortality rates between Cohort A and Cohort B.

	Cohort B (January 2021-March 2022)													Cohort A (March-July 2020, described in reference 1)										
	n	Age(yr, IQR)	Sex (n, % male)	IgRT(n, %)	pAbx (n, %)	IS(n, %)	Hospital-ised(n, %)	Deaths (n)	Inpatient mortality (%)	IFR (%)	n	Age (yr, IQR)	Sex(n, % male)		IgRT (n, %)	pAbx(n, %)	IS(n, %)	Hospital-ised(n, %)	Deaths(n)	Inpatient mortality (%)	IFR (%)	p-value (hospital-isation)	p value (inpatient mortality)	p value (IFR)
**Primary Immunodeficiency (all)**	**117**	**45** **(33-59)**	**62 (52.9)**	**98** **(83.8)**	**60 (51.2)**	**19 (16.2)**	**21** **(17.9)**	**4**	**19.0**	**3.42**	60	42.0 (28.0-58.2)	26 (43.3)		42 (70.0)	32 (53.3)	11 (18.3)	32 (53.3)	12	37.5	20.0	**< 0.0001**	**0.15**	**0.0003**
Primary Immunodeficiency (untreated, no prior COVID-19, 2x vaccinated)	26	45 (35-61)	13 (50.0)	23 (88.4)	15 (57.6)	7 (26.9)	0 (0.0)	0	0.0	0.0												**<0.0001**	**-**	**0.01**
**Common variable immunodeficiency** **(all)**	**50**	**45** **(37-60)**	**24 (48.0)**	**48** **(96.0)**	**29 (58.0)**	**11 (22.0)**	**10** **(20.0)**	**1**	**10.0**	**2.0**	23	54.0 (31.8-70.8)	9 (39.1)		20 (87.0)	11 (47.8)	4 (17.4)	13 (56.5)	8	61.5	34.8	**0.002**	**0.01**	**<0.0001**
CVID (untreated, no prior COVID-19, 2x vaccinated)	13	44 (35-53)	6 (46.2)	13 (100.0)	8 (61.5)	3 (23.1)	0 (0.0)	0	0.0	0.0												**0.0007**	**-**	**0.02**
**Secondary Immunodeficiency (all)**	**38**	**57** **(48-65)**	**17 (44.7)**	**27** **(71.1)**	**19 (50.0)**	**14 (36.8)**	**7** **(18.4)**	**3**	**42.8**	**7.89**	33	64.5 (56.0-79.8)	15 (45.5)		20 (87.0)	25 (75.8)	12 (36.3)	25 (75.8)	11	44.0	33.3	**< 0.0001**	**0.96**	**0.007**
Secondary Immunodeficiency (untreated, no prior COVID-19, 2x vaccinated)	13	59 (44-63)	6 (46.1)	8 (61.5)	6 (46.1)	6 (46.1)	0 (0.0)	0	0.0	0.0												**<0.0001**	**-**	**0.02**

For continuous variables the median and interquartile range is provided. Categorical variables are compared using the Chi-square test. IgRT – immunoglobulin replacement therapy, pAbx – prophylactic antibiotics, IS – current immune suppression, IFR – infection-fatality ratio, CVID – common variable immunodeficiency, COVID-19 – Coronavirus disease-19. Untreated refers to individuals who received no specific inpatient or outpatient treatment for COVID-19 (i.e. antivirals, monoclonal antibodies, steroids or biologic therapies). In bold to draw attention to them, they are primary headings for the tables.

**Table 3:** Narrative review of deaths in Cohort B.

Patient	Age	Sex	Diagnosis	IgRT	pABX	Immunosuppression	Prior COVID-19	Comorbidities	Vaccines received at point of infection + serological response	SARS-CoV-2 variant	Treatment and notes
1	68	F	SID	Y	Y	Rituximab, prednisolone	N	CVD, inflammatory arthritis	2 Negative	Delta	Disease complicated by severe pneumonitis and cardiac arrythmias. Received dexamethasone and remdesvir. Previously described in (10)
2	45	F	SID	Y	N	Prednisolone, HCQ	N	CVD, SLE, CKD	2 Positive	Delta	Received inpatient Ronapreve (casirivimab/imdevimab)
3	23	M	22q11	N	Y	N	N	CVD	2 Positive	Delta	Received dexamethasone and remdesivir, CPAP and mechanical ventilation but died.
4	87	M	CVID	N	Y	N	N	CVD, bronchiectasis,	3 Negative	Omicron	Initially vague COVID-19 symptoms so no outpatient based treatment. Received dexamethasone as inpatient.
5	53	M	CID	Y	N	N	N	CVD, recurrent pneumothoraces, granulomatous disease of liver and bone marrow, hepatocellular carcinoma, CKD, pancytopaenia	3 Negative	Delta	COVID pneumonitis, treated with ronapreve, remdesivir and dexamethasone, complicated by community acquired pneumonia and respiratory failure worsened by gross ascites secondary to hepatocellular carcinoma
6	56	F	SID	Y	Y	Steroids, azathioprine, belatacept	N	CVD, CKD, Renal transplant	2 Negative	Omicron	Received sotrovomab late in disease course as PCR returned positive result 6d before symptom onset.
7	77	M	PAD	Y	Y	Prednisolone	N	CVD, CKD, COPD	3 Unknown	Omicron	Received molnupiravir, but required subsequent admission.

CVD, cardiovascular disease; CID, combined immunodeficiency; CKD, chronic kidney disease; COPD, chronic obstructive pulmonary disease; SLE, systemic lupus erythematosus; ICU, intensive care unit; HCQ, hydroxychloroquine; CPAP, continuous positive airway pressure; PCR, polymerase chain reaction; IgRT, immunoglobulin replacement therapy; pAbx, prophylactic antibiotics.

To isolate the effect of vaccination on outcome, Cohort A (SARS-CoV-2 immunologically naive, no specific treatment, unvaccinated) was compared with individuals in Cohort B infected for the first time after receipt of two vaccine doses but receiving no pharmacological treatment for COVID-19 (**Table 2**). In this subgroup analysis, statistically significant reductions in hospitalization and IFR were also observed in PID, CVID and SID, providing further evidence of vaccine efficacy in individuals with immunodeficiency.

It has been postulated that the Omicron variant, that emerged as the dominant UK strain in December 2021, is more transmissible but less virulent than previous SARS-CoV-2 variants (19). To consider this in the context of immunodeficiency we compared the outcomes of individuals in Cohort B infected with Omicron to those infected with prior variants, based on timing of infection with reference to longitudinal UKHSA sequencing data documenting the dominant circulating variant throughout the pandemic (13). Hospitalization rates were significantly lower (41.5% vs 9.9%, p<0.0001) and the infection fatality rate lower (9.8% vs 2.7%, p=0.07) following infection with Omicron, compared to prior variants. However, by the time of infection with Omicron, individuals had had the opportunity to receive significantly more vaccine doses (1.9 doses vs 3.0 doses, p<0.0001) leading to a greater percentage having anti-SARS-CoV-2 antibodies at the time of infection (62.3% vs. 19.4%, p=0.0001). This is in keeping with data demonstrating enhanced seroprevalence following a third vaccine dose in patients with antibody deficiency (10). However, the hospitalization rate (9.9% vs 2.2%, p<0.0001) and IFR (2.7% vs 0.2%, p<0.0001) have remained significantly higher in immunodeficiency patients than the general population during the Omicron wave.

Anti-SARS-CoV-2 spike antibody levels were available for 84 individuals following vaccination but prior to SARS-CoV-2 infection: to understand the importance of these antibodies, we compared the outcomes of individuals with and without a serological response to prior vaccination. Individuals with evidence of a serological response to vaccination at the time of infection had received a significantly greater number of vaccine doses and were more likely to have been infected during the Omicron wave (**Table 4**). However, no significant differences were observed in the hospitalization rate (26.7% vs 15.3%) or mortality rates (8.9% vs 5.1) of individuals based on their serostatus at the time of infection in this study.

**Table 4:** Comparison of hospitalization and mortality rates in individuals based on antibody response to prior vaccination.

	No antibody response (n = 45)	Antibody response (n = 39)
Age (median, IQR)	52 (39.0-66.0)	51 (36.0-64.0)
Sex (n, % male)	29 (64.4)	16 (41.0)
Prior COVID (n, %)	5 (11.1)	4 (10.2)
2 vaccine doses (n, %)	43 (95.6)	39 (100.0)
3 vaccine doses (n, %)	35 (77.8)	36 (92.3)
Mean vaccine doses received at time of infection	2.5	2.9 *
Infection with Omicron variant (n, %)	20 (44.4)	33 (84.6) **
Immunoglobulin replacement therapy (n, %)	40 (88.9)	34 (87.2)
Prophylactic antibiotics (n, %)	26 (57.8)	18 (46.2)
Immunosuppression (n, %)	12 (26.7)	8 (20.5)
Symptomatic (n, %)	39 (86.7)	34 (87.1)
Received treatment (n, %)	27 (60.0)	26 (66.7)
Hospitalised (n, %)	12 (26.7)	6 (15.4)
Deaths	4	3
**IFR (%)**	**8.9**	**7.7**

Categorical variables are compared using the Chi-square test. * denotes p<0.0001, ** denotes p=0.0001.

As part of the COV-AD study, participants were sent serial swabs every two weeks after infection until viral clearance was demonstrated. Individuals with a serological response to prior vaccination demonstrated more rapid viral clearance from the nasopharynx (27.0 days vs 55.5 days, p=0.03) (**Figure 1D**) with a trend towards more rapid clearance in sputum samples (40.0 days vs 58.0 days, p=0.06) where viral RNA can be detected for significantly longer than the nasopharynx (24.0 days vs 42.5 days, p<0.00001) (**Figure 1E**). Two individuals demonstrated prolonged nasopharyngeal carriage of over 200 days; both were infected prior to the widespread availability of outpatient treatments for SARS-CoV-2. Patient A was a 30-year-old individual with CVID complicated by autoimmune liver disease and an inflammatory arthropathy infected shortly after treatment with rituximab and prior to vaccination. The patient demonstrated strong T cell responses by ELISPOT to peptide pools derived from both the SARS-CoV-2 spike and nucleocapsid proteins; 2 doses of the AstraZeneca vaccine administered during ongoing viral infection failed to achieve viral clearance. Instead, PCR negativity was temporally associated with the re-emergence of a peripheral B cell population, but an antibody response only became detectable following third primary immunization 4 months later. Patient B was a 46-year-old individual with CVID complicated by bronchiectasis, splenomegaly, and chronic norovirus infection. They were receiving no exogenous immunosuppression, had detectable T cell responses to viral spike and nucleocapsid peptide pools 1 month after infection, but no detectable antibody response after three vaccinations.

Overall, 63.2% (n=98/155) of individuals in this cohort received specific treatment for COVID-19; 73 as an outpatient and 25 as an inpatient (**Table 5**). Acute outpatient treatment of COVID-19 for immunodeficient individuals using oral antivirals or monoclonal antibodies within 5 days of symptom onset became available after the 16^th^ December 2021, through NHS COVID-19 Medicine Delivery Units (CMDU). 61.4% (n=70/114) of eligible patients infected after this date were treated by CMDU, most commonly with sotrovimab (n=38/70) or molnupiravir (n=16/70). Concordant with other studies (21), we found significantly lower rates of hospitalization (4.3% vs 15.9%, p=0.03) amongst individuals treated by CMDU but overall mortality was not affected (2.8% vs 4.5%, p=0.63). Due to the range of different treatment combinations used and small numbers, it was not possible to draw conclusions on the most effective therapeutic to prevent hospitalization in this cohort.

**Table 5:** Treatments following SARS-CoV-2 infection received by participants in Cohort B in outpatient and inpatient settings.

Treatment	N	Deaths
*Outpatient*	73	0
Sotrovimab	38	0*
Molnupiravir	17	0*
Paxlovid	10	0
Remdesivir	2	0
Antiviral not specified	6	0
*Inpatient*	25	6*
Ronapreve monotherapy	5	1
Remdesivir, dexamethasone and Ronapreve	3	1
Remdesivir and Ronapreve	3	0
Remdesivir and Sotrovimab	3	0
Remdesivir and dexamethasone	2	2
Sotrovimab monotherapy	2	0
Dexamethasone monotherapy	1	1
Remdesivir, dexamethasone, Ronapreve, sarilumab	1	0
Remdesivir, dexamethasone, Ronapreve, tocilizumab	1	0
Remdesivir monotherapy	1	0
Ronapreve and dexamethasone	1	0
Ronapreve, dexamethasone and sarilumab	1	1
Monoclonal antibody not otherwise specified	1	0
No further treatment	1	1

* - one individual received sotrovimab late in disease course and one individual molnupiravir prior to subsequent hospital admission and death.

## Discussion

Outcomes following SARS-CoV-2 infection in patients with PID and SID have greatly improved since the start COVID-19 pandemic, but remain significantly worse than the general population. The reasons for these improvements are likely to be multifactorial: non-pharmacological interventions (e.g. legal restrictions on social interactions, mandated face coverings in public), vaccination, therapeutics, and the emergence of potentially less virulent SARS-CoV-2 variants may all contribute to the observed reduction in hospitalization and mortality in patients with PID and SID. Dissecting the impact of any individual factor on SARS-CoV-2 outcome is challenging given the rapid evolutionary pace of the pandemic and simultaneous deployment of multiple public health interventions. Although our analyses have limitations, including the comparison of prospectively collected data with a published, retrospectively gathered historic cohort, we believe that these data provide important insights into the burden of SARS-CoV-2 infection on patients with PID and SID as the pandemic progresses towards endemicity.

Firstly, by leveraging the COV-AD study which enrolled a cohort of over 500 individuals with antibody deficiency, followed longitudinally through the pandemic, we demonstrate that over three-quarters of individuals with antibody deficiency have not yet been knowingly infected with the SARS-CoV-2 virus. The significant risk that SARS-CoV-2 infection posed to adult patients with PID and SID became clear during the first wave of the UK pandemic (1) and led to the PID and SID communities stringent adherence to preventative non-pharmacological interventions (22). Asymptomatic SARS-CoV-2 infection is unusual in individuals with immunodeficiency: no patients enrolled in COV-AD tested asymptomatically positive by PCR at study entry and over 80% of individuals testing positive for SARS-CoV-2 in this cohort study demonstrate symptoms of COVID-19. These data suggest that there remains a large cohort of patients with PID or SID who have yet to experience SARS-CoV-2 infection.

When compared to the general population, non-pharmacological measures appear to have delayed SARS-CoV-2 infections in individuals with immunodeficiency until the Omicron wave, by which time, most had received a third-primary vaccination and were more likely to have some humoral immunity against SARS-CoV-2, evidenced by higher seroprevalence of anti-SARS-CoV-2 spike antibodies. Although significant improvements in morbidity and mortality have been realized compared to the initial wave of the SARS-CoV-2 pandemic, individuals with immunodeficiency remain at significantly increased risk of poor outcome compared to the general population, with an IFR of 2.7% during the Omicron wave. Thus, SARS-CoV-2 continues to pose a serious risk to these patients. We provide evidence that early outpatient treatment mitigates some of this risk but given less than two-thirds of individuals eligible for early treatment received it, access does not appear to be universal and fatalities have occurred despite vaccination and early treatment. Prophylactic monoclonal antibodies may provide an additional protective layer for patients with immunodeficiency.

Our data strongly suggests that vaccination affords significant real-world protection against hospitalization and death in patients with immunodeficiency. Yet, the immunological mechanisms underlying this protection remain unclear. The presence of a serological responses to vaccination was associated with a shorter duration of SARS-CoV-2 viral RNA carriage in the nasopharynx, but no significant effect was observed on the risk of hospitalization or death following SARS-CoV-2 infection. The presence of neutralizing antibodies remains the best understood correlate of protection against infection and severe disease arising from vaccine studies (23), and effective, humoral immunity has previously been demonstrated to be non-redundant to achieving viral clearance in some patients with immunodeficiency (24, 25). The lack of a strong effect of antibody on outcome within this study may be due to heterogeneity in the magnitude of the serological response to vaccination (26) and the diminished cross-reactivity of antibodies raised against Wuhan-variant vaccines against novel variants of concern (9, 10), especially given the proportion of individuals infected with Omicron in this study. Although serological responses can be boosted by additional vaccination in patients with antibody deficiency (10), high titre, prophylactic neutralizing monoclonal antibodies may provide superior and more predictable real-world protection in these individuals (14). Moving forward, the immunological response to variant-specific vaccines must be studied in patients and the potency of prophylactic and therapeutic neutralizing monoclonal antibodies against emerging variants of concern carefully monitored to ensure ongoing efficacy.

In conclusion, we report the epidemiology of the ongoing SARS-CoV-2 pandemic in individuals with PID and SID in the United Kingdom and provide evidence of the efficacy of vaccination and early therapeutics in reducing hospitalization and mortality rates following infection. SARS-CoV-2 continues to pose a significant risk to patients with PID and SID and there remains an unmet need to understand the correlates of protection against severe disease and optimize mitigation, prophylactic and therapeutic strategies to minimize the ongoing burden of the pandemic to these vulnerable groups.

## Data availability statement

The raw data supporting the conclusions of this article will be made available by the authors, without undue reservation.

## Ethics statement

The studies involving human participants were reviewed and approved by London - Dulwich Research Ethics Committee (REC reference: 21/LO/0162). The patients/participants provided their written informed consent to participate in this study.

## The COV-AD consortium

The following Authors, who are listed in alphabetical order, contributed to the work of the COV-AD consortium:

**Zahra Ahmed,** Clinical Immunology Service, Institute for Immunology and Immunotherapy, University of Birmingham, United Kingdom; **Fiona Ashford,** Clinical Immunology Service, Institute for Immunology and Immunotherapy, University of Birmingham, United Kingdom; **Hollie Bancroft, ** University Hospitals Birmingham NHS Foundation Trust, Birmingham, United Kingdom; **Michelle Bates,** University Hospitals Birmingham NHS Foundation Trust, Birmingham, United Kingdom; **Angus Best,** Clinical Immunology Service, Institute for Immunology and Immunotherapy, University of Birmingham, United Kingdom; **Naomi Campton,** Institute of Translational Medicine, University of Birmingham, Birmingham, United Kingdom; **Michele Chan,** Department of Immunology, Royal Free London NHS Foundation Trust, London, United Kingdom; **Samuel Chee,** Department of Immunology and Allergy, University Hospital Plymouth NHS Trust, Plymouth, United Kingdom; **Hayley Clifford,** University Hospitals Birmingham NHS Foundation Trust, Birmingham, United Kingdom; **Lucy Common,** Department of Immunology and Allergy, University Hospital Plymouth NHS Trust, Plymouth, United Kingdom; **Georgina Davis,** Department of Immunology, Salford Royal NHS Foundation Trust, Salford, United Kingdom; **Joanne Dasgin,** Clinical Immunology Service, Institute for Immunology and Immunotherapy, University of Birmingham, United Kingdom; **Mohammad Dinally,** Clinical Immunology Service, Institute for Immunology and Immunotherapy, University of Birmingham, United Kingdom; **Fatima Dhalla**, Department of Clinical Immunology, Oxford University Hospitals NHS Foundation Trust, Oxford, United Kingdom; **Elena Efstathiou**, Clinical Immunology Service, Institute for Immunology and Immunotherapy, University of Birmingham, United Kingdom; **Sian Faustini**, Clinical Immunology Service, Institute for Immunology and Immunotherapy, University of Birmingham, United Kingdom; **Mark Gompels**, Department of Immunology, North Bristol NHS Trust, Bristol, United Kingdom; **Dan Hartland**, Saving Lives Charity, MIDRU Building, Heartlands Hospital, Birmingham, United Kingdom; **Archana Herwadkar**, Department of Immunology and Allergy, University Hospital Plymouth NHS Trust, Plymouth, United Kingdom; **Harriet Hill**, Institute for Immunology and Immunotherapy, University of Birmingham, Birmingham, United Kingdom; **Madeeha Hoque**, Clinical Immunology Service, Institute for Immunology and Immunotherapy, University of Birmingham, United Kingdom; **Emily Heritage**, Institute of Translational Medicine, University of Birmingham, Birmingham, United Kingdom; **Gail Heritage**, Institute of Translational Medicine, University of Birmingham, Birmingham, United Kingdom; **Deborah Hughes**, Department of Immunology, University Hospital North Midlands, Stoke, United Kingdom; **Joe Humphreys**, Institute of Translational Medicine, University of Birmingham, Birmingham, United Kingdom; **Ann Ivory**, Department of Immunology, University Hospital North Midlands, Stoke, United Kingdom; **Rashmi Jain**, Department of Clinical Immunology, Oxford University Hospitals NHS Foundation Trust, Oxford, United Kingdom; **Sarah Johnston**, Department of Immunology, North Bristol NHS Trust, Bristol, United Kingdom; **Sinead Kelly**, Newcastle upon Tyne Hospitals NHS Foundation Trust, Newcastle upon Tyne, Newcastle, United Kingdom; **Karen Knowles**, Department of Immunology and Allergy, University Hospital Plymouth NHS Trust, Plymouth, United Kingdom; **Me Me Nay Lin**, Department of Immunology, Royal Free London NHS Foundation Trust, London, United Kingdom; **Fernando Moreira**, Department of Immunology, Royal Free London NHS Foundation Trust, London, United Kingdom; **Theresa McCarthy**, Clinical Immunology Service, Institute for Immunology and Immunotherapy, University of Birmingham, United Kingdom; **Christopher McGee**, University Hospitals Birmingham NHS Foundation Trust, Birmingham, United Kingdom; **Daniel Mullan**, Department of Immunology, Salford Royal NHS Foundation Trust, Salford, United Kingdom; **Hadeil Morsi**, Department of Clinical Immunology, Oxford University Hospitals NHS Foundation Trust, Oxford, United Kingdom; **Eileen O’Grady**, Department of Allergy and Clinical Immunology, Leeds Teaching Hospitals NHS Trust, Leeds, United Kingdom; **Thomas O’Hagan**, Department of Immunology, Royal Free London NHS Foundation Trust, London, United Kingdom; **Shannon Page**, Clinical Immunology Service, Institute for Immunology and Immunotherapy, University of Birmingham, United Kingdom; **Nicholas Peters**, Department of Clinical Immunology, Oxford University Hospitals NHS Foundation Trust, Oxford, United Kingdom; **Timothy Plant**, Clinical Immunology Service, Institute for Immunology and Immunotherapy, University of Birmingham, United Kingdom; **Maria Poulaka**, Department of Immunology and Allergy, University Hospital Plymouth NHS Trust, Plymouth, United Kingdom; **Archana Shajidevadas**, Research and Development Department, University Hospital Plymouth NHS Trust, Plymouth, United Kingdom; **Malgorzata Slowinsksa**, Department of Immunology, North Bristol NHS Trust, Bristol, United Kingdom; **Zania Stamataki**, Institute for Immunology and Immunotherapy, University of Birmingham, Birmingham, United Kingdom; **Zehra Suleiman**, Clinical Immunology Service, Institute for Immunology and Immunotherapy, University of Birmingham, United Kingdom; **Chloe Tanner**, Clinical Immunology Service, Institute for Immunology and Immunotherapy, University of Birmingham, United Kingdom; **Neil Townsend**, Clinical Immunology Service, Institute for Immunology and Immunotherapy, University of Birmingham, United Kingdom; **Charlotte Trinham**, Clinical Immunology Service, Institute for Immunology and Immunotherapy, University of Birmingham, United Kingdom; **Stuart Wareham**, Department of Immunology, Salford Royal NHS Foundation Trust, Salford, United Kingdom; **Sinead Walder**, Clinical Immunology Service, Institute for Immunology and Immunotherapy, University of Birmingham, United Kingdom; **Hollie Wagg**, Institute of Translational Medicine, University of Birmingham, Birmingham, United Kingdom; **Sarita Workman**, Department of Immunology, Royal Free London NHS Foundation Trust, London, United Kingdom.

## Author contributions

AMS, SS, DL, SOB and AGR designed and supervised the study. JEDT, PK, SOB and AGR provided senior leadership and strategic oversight for the COVAD study. ST, AAH, JMH, MMNL, MC, SG, JD, MD, SYP, SE, AH, CJAD, AH, SK, CB and ShE and SuE recruited patients to the study. AMS analysed the data, wrote the first draft of the manuscript and revised the manuscript. All authors contributed the revision of the manuscript and read and approved the final version.

## Funding

This study was funded by United Kingdom Research and Innovation (MR/W002663/1).

## Acknowledgments

The authors are grateful to the UK Primary Immunodeficiency Network (UK PIN), its members, the COV-AD study participants, Oxford Immunotec, and the Immunodeficiency patient community within the United Kingdom for their support of this study.

## Conflict of interest

The authors declare that the research was conducted in the absence of any commercial or financial relationships that could be construed as a potential conflict of interest.

## Publisher’s note

All claims expressed in this article are solely those of the authors and do not necessarily represent those of their affiliated organizations, or those of the publisher, the editors and the reviewers. Any product that may be evaluated in this article, or claim that may be made by its manufacturer, is not guaranteed or endorsed by the publisher.

## Abbreviations

COV-AD, Coronavirus in patients with antibody deficiency; COVID-19, Coronavirus disease 2019; CTLA-4, Cytotoxic T-lymphocyte-associated protein 4; CVID, Common variable immunodeficiency; ICOS, Inducible T-cell Costimulator; IgRT, Immunoglobulin replacement therapy; FR, Infection fatality ratio; IPEX, Immunodysregulation polyendocrinopathy enteropathy X-linked; IRAK-4, Interleukin-1 receptor-associated kinase 4; NFB1, Nuclear Factor Kappa B Subunit 1; pAbx, Prophylactic antibiotics; PID, Primary immunodeficiency; PNP SCID, Purine nucleoside phosphorylase severe combined immunodeficiency syndrome; SARS-CoV-2, Severe acute respiratory syndrome coronavirus 2; SID, Secondary immunodeficiency; STAT, Signal transducer and activator of transcription; UKPIN, United Kingdom Primary Immunodeficiency Network.
